# Factors contributing to diagnostic delay of Caroli syndrome: a single-center, retrospective study

**DOI:** 10.1186/s12876-020-01442-5

**Published:** 2020-09-29

**Authors:** Wen Shi, Xiao-ming Huang, Yun-lu Feng, Feng-dan Wang, Xiao-xing Gao, Yang Jiao

**Affiliations:** 1 Department of Gastroenterology, Peking Union Medical College Hospital, Chinese Academy of Medical Sciences & Peking Union Medical College, Beijing, China; 2 Department of General Internal Medicine, Peking Union Medical College Hospital, Chinese Academy of Medical Sciences & Peking Union Medical College, No. 1, Shuaifuyuan, Wangfujing St., Beijing, 100730 China; 3 Department of Radiology, Peking Union Medical College Hospital, Chinese Academy of Medical Sciences & Peking Union Medical College, Beijing, China

**Keywords:** Caroli syndrome, Diagnosis, Imaging

## Abstract

**Background:**

Caroli syndrome (CS) is a rare congenital disorder without pathognomonic clinical symptoms or laboratory findings; therefore, the diagnosis is often delayed. The objective of this study was to investigate the diagnostic delay and associated risk factors in CS patients.

**Methods:**

This was a retrospective analysis of 16 CS patients admitted to a single tertiary medical center on mainland China. The diagnostic timelines of CS patients were reviewed to demonstrate the initial findings of CS at diagnosis, the risk factors associated with diagnostic delay, and potential clues leading to early diagnosis.

**Results:**

The median diagnostic delay was 1.75 years (range: 1 month to 29 years, interquartile range: 6.2 years) in 16 enrolled CS patients. Sex, age, and initial symptoms were not associated with diagnostic delay. 87.5% of CS patients were diagnosed by imaging, and the accuracies of ultrasonography, computed tomography (CT), and magnetic resonance cholangiopancreatography were 25, 69.2, and 83.3%, respectively. The median diagnostic delays for patients with or without CT performed at the first hospital visited according to physician and radiologist suspicion of the diagnosis were 7.4 months and 6 years, respectively (*p* = 0.021). Hepatic cysts with splenomegaly were detected by ultrasound in over half of CS patients.

**Conclusions:**

The majority of CS patients were not diagnosed until complications of portal hypertension had already developed. Recognition and early suspicion of the disease were important factors influencing diagnostic delay of CS. Hepatic cysts plus splenomegaly detected by US might raise the clinical suspicion to include CS in the differential diagnosis.

## Background

Caroli syndrome (CS) is a rare congenital disorder characterized by segmental dilatation of the intrahepatic ducts and hepatic fibrosis [1]. The incidence of CS is estimated to be 1 per million of the population [2]. Although the pathoetiology of CS is still poorly understood, CS is known to be an autosomal recessive hereditary disorder involving malformation of the ductal plates and associated periportal fibrosis [3].

CS has no pathognomonic clinical symptoms or signs [4]. It can manifest insidiously, with patients presenting in two main ways: intrahepatic ductal ectasia and bile stagnation (i.e., recurrent cholangitis and/or cholangiolithiasis) or portal hypertension (i.e., hypersplenism, gastrointestinal bleeding, ascites) [5]. CS has also been reported in association with cystic renal disease, pancreatic cysts, cavernomatous transformation of the portal vein, and an increased risk of cholangiocarcinoma [6].

Histopathology is useful for securing a definitive diagnosis, but imaging modalities including ultrasonography (US), computed tomography (CT), and magnetic resonance imaging (MRI) remain the first-line diagnostic methods due to their noninvasiveness and convenience [7]. Although an early diagnosis of CS is the first step to early intervention, complication control, and surveillance, there is scarce evidence on the clinical parameters that influence early diagnosis or how imaging and their features might influence the diagnostic timeline of CS patients [8].

We therefore performed a single-center retrospective study to review the diagnostic timelines of 16 CS patients to investigate the clinical features at diagnosis, the risk factors associated with diagnostic delay, and potential clues leading to early diagnosis.

## Methods

Sixteen CS patients were admitted to Peking Union Medical College Hospital between January 12,005 and August 12,019. Demographic data, symptoms, laboratory results, detailed imaging findings, associated conditions, histopathology results, and other information related to CS diagnosis including residential location, family history, and hospitals visited were collected from the medical records. A diagnostic timeline was drawn for each patient using these data. We defined diagnostic delay as the time between initial clinical presentation and final diagnosis.

Data were analyzed using SPSS for Windows Version 23.0 (IBM Statistics Inc., Chicago, IL). Continuous variables were reported as means ± standard deviation (SD) and compared using Student’s *t*-test if determined as normally distributed according to the Shapiro-Wilk test. Otherwise, they were reported as median values with interquartile ranges (IQR) and compared using the Mann-Whitney U test. Categorical variables were analyzed using the chi-squared test. Both continuous and categorical variables were analyzed using logistic regression or Spearman’s coefficient. A *p*-value < 0.05 was considered statistically significant. Since diagnostic delay was not normally distributed, we took the natural logarithm (ln) of the diagnostic delay (ln (diagnostic delay)), which was normally distributed, which allowed the use of *t*-tests with this variable.

## Results

### Demographic features

Ten CS patients were male and six were female, with a male to female ratio of 5:3. The median age of symptom onset was 7 years of age (range: 1 month to 38 years, IQR: 15.5 years), the median age of first clinic visit was 10 years of age (range: 1 month to 38 years, IQR: 17.6 years), and the median age at diagnosis was 15 years of age (range: 8 months to 39 years, IQR: 24.5 years). The median diagnostic delay, which was defined as the time between initial clinical presentation and final diagnosis, was 1.75 years (range: 1 month to 29 years, IQR: 6.2 years). Sex, age of symptom onset, age at first clinic visit, and age at diagnosis were not significantly associated with diagnostic delay (Table 1).

**Table 1:** Association between ln (diagnostic delay) and demographics, initial symptoms, laboratory findings, and image modalities used

	N	Mean	SD	*P*-value
Ln (DD)				
Male	10	0.76	1.74	
Female	6	0.17	2.20	*p* = 0.56
^a^Age of onset of symptoms				*p* = 0.52
^a^Age of first clinical visit				*p* = 0.99
^a^Age of diagnosis				*p* = 0.17
^b^Fever	6	0.41	2.31	
Not fever	10	0.61	1.70	*p* = 0.84
^b^Abdominal pain	3	0.63	1.51	
Not abdominal pain	13	0.52	2.00	*p* = 0.93
^b^Abdominal distention	8	0.84	1.39	
Not abdominal distention	8	0.23	2.32	*p* = 0.54
Normal WBC	5	−1.47	1.36	
Decreased WBC	11	1.45	1.27	*p = 0.01*
Normal HGB	5	−0.47	1.57	
Decreased HGB	11	0.99	1.89	*p* = 0.16
Normal PLT	6	−0.92	1.80	
Decreased PLT	10	1.41	1.34	*p = 0.01*
No pancytopenia	6	−0.92	1.80	
Pancytopenia	10	1.41	1.34	*p = 0.01*
Normal PT	11	0.51	2.16	
Prolonged PT	5	0.59	1.25	*p* = 0.94
CT at first hospital visited	8	−0.55	1.76	
No CT at first hospital visited	8	1.62	1.33	*p = 0.02*

Initial clinical presentations of gastrointestinal bleeding or fatigue, elevated alanine aminotransferase or bilirubin, decreased albumin, positive antinuclear antibody, anti-smooth muscle antibodies, or anti-mitochondrial antibodies M2 subtype are not analyzed due to the small sample size (*n* < 3)

*Ln* Natural logarithm, *DD* Diagnostic delay, *WBC* White blood cell, *HGB* Hemoglobin, *PLT* Platelet, *PT* Prothrombin time, *CT* Computed tomography, *SD* Standard deviation

^a^ Calculated and tested by Pearson correlation coefficient

^b^ Initial clinical presentations

### Initial clinical presentation

Initial symptoms of the 16 CS patients included fever (*n* = 6), abdominal pain (*n* = 3), abdominal distention (*n* = 8), gastrointestinal bleeding (*n* = 1), and fatigue (*n* = 1). Most initial symptoms led directly to a first clinic visit, with the exception of three patients who had abdominal distention as the initial symptom but only presented later to hospital for other reasons (Table 2). None of these initial symptoms were associated with diagnostic delay (Table 1). Three patients had a positive family history, but similarly this was not associated with diagnostic delay (Table 1).

**Table 2:** Initial clinical presentations of CS patients

	Initial clinical presentation Patients (%)	Initial clinical presentation leading to first clinical visit Patients (%^a^)
Fever	6 (37.5%)	6 (100%)
Fever + abdominal pain	3 (18.75%)	3 (100%)
Abdominal distention	8 (66.7%)	5 (62.5%)
Fever + abdominal distention	1 (6.25%)	1 (100%)
Gastrointestinal bleeding	1 (6.25%)	1 (100%)
Fatigue	1 (6.25%)	1 (100%)
Other	1 (6.25%)^b^	

*CS* Caroli syndrome

^a^ % of patients in whom the very initial clinical presentation led to the first clinical visit

^b^ The patient went to hospital due to upper respiratory infection and was found to have splenomegaly

### Laboratory findings

Laboratory findings at diagnosis included decreased peripheral white blood cell count (*n* = 11), anemia (*n* = 11), and decreased platelet count (*n* = 10). Ten patients had pancytopenia. Some patients had abnormal liver function, with elevated serum alanine aminotransferase (ALT) (*n* = 2), elevated serum bilirubin (*n* = 2), decreased serum albumin (*n* = 2), and prolonged prothrombin time (PT) (*n* = 5). Of note, the two patients with elevated ALT had cholangitis when admitted, and their ALT levels returned to normal after anti-microbial treatment. Two patients had increased serum creatinine levels, both of whom had multiple renal cysts.

Positive autoantibodies were present in four CS patients: antinuclear antibodies (ANA) were tested in ten patients and two were positive; anti-mitochondrial antibodies (M2 subtype; AMA-M2) were tested in seven patients and two were positive; and anti-smooth muscle antibodies (SMA) were tested in seven patients and one was positive. Two patients were initially misdiagnosed as having autoimmune disease. The first misdiagnosed patient was a 21-year-old man with positive ANA and slightly elevated aspartate transaminase (AST) and immunoglobulin G (IgG), who was misdiagnosed with autoimmune hepatitis (AIH) and was treated with corticosteroids and azathioprine for 1 month. There was no clinical improvement, and histopathological examination of his liver biopsy secured a diagnosis of CS. The other misdiagnosed patient was a 33-year-old woman who presented with low-grade fever and abdominal distention who had positive ANA and AMA-M2 and slightly elevated gamma-glutamyltransferase (GGT) and alkaline phosphatase (ALP). She was initially misdiagnosed with primary biliary cholangitis (PBC) and treated with corticosteroids and ursodeoxycholic acid for 3 months. Again, there was no clinical improvement and she developed recurrent cholangitis. Magnetic resonance cholangiopancreatography (MRCP) was performed, and the diagnosis of CS was made according to typical imaging findings. Her symptoms were soon controlled with antibiotics, and her GGT and ALP returned to normal.

Lower white blood cell (WBC) counts (5 years vs. 4 months; *p* = 0.01), lower platelet (PLT) counts (4.5 years vs. 6.5 months; *p* = 0.01), and pancytopenia (4.5 years vs. 6.5 months; *p* = 0.01) at diagnosis were associated with longer diagnostic delay. There were no other significant associations between laboratory findings and diagnostic delay (Table 1).

### Imaging manifestations

All 16 CS patients were examined by US before diagnosis, 13 were examined by CT with or without contrast, and 12 were investigated by MRCP. Some patients had several imaging studies at different hospitals before the final diagnosis was secured. On average, US was performed 1.9 times and CT 1.3 times per patient before diagnosis. The accuracies of US, CT, and MRCP were 25, 69.2, and 83.3%, respectively (Table 3). Fourteen patients were diagnosed by imaging, while two patients were finally diagnosed from the histopathological appearances on liver biopsy.

**Table 3:** Imaging studies of CS patients

Image modality	Patients examined before diagnosis	Average times examined per patient	Patients diagnosed by this modality	Accuracy
US	16	1.9	4	25%
CT	13	1.3	9	69.2%
MRCP	12	0.8	10	83.3%
Any imaging	16	4.4	14	87.5%

*CS* Caroli syndrome, *CT* Computed tomography, *MRCP* Magnetic resonance cholangiopancreatography, *US* Ultrasound

All 16 patients had US performed during the first visit to a hospital, while eight patients also had CT scans due to suspicions raised by physicians and radiologists. CT performed at the first hospital visited was associated with a statistically significant shorter diagnostic delay (*p* = 0.021; Table 1). The median diagnostic delays for patients with CT performed or not performed at the first hospital visit were 7.4 months and 6 years, respectively.

We examined the US signs in CS patients at their first hospital visit. Diffusive hepatic lesions, hepatic cysts, splenomegaly, and renal cysts were reported in 10 (62.5%), 10 (62.5%), 15 (93.8%), and 8 (50%) patients, respectively. The combination of diffuse hepatic lesions plus splenomegaly, hepatic cysts plus splenomegaly, and renal cysts plus splenomegaly were found in 10 (62.5%), 9 (56.3%), and 8 (50%) patients, respectively (Table 4).

**Table 4:** Sensitivity of different signs by US in diagnosing CS patients

	Patients/Patients tested	Proportion
Splenomegaly	15/16	93.8%
Diffusive hepatic lesions	10/16	62.5%
Hepatic cysts	10/16	62.5%
Diffusive hepatic lesions + splenomegaly	10/16	62.5%
Hepatic cysts + splenomegaly	9/16	56.3%
Renal cysts	8/16	50%
Renal cysts + splenomegaly	8/16	50%
Hepatic cysts + renal cysts + splenomegaly	6/16	37.5%

*CS* Caroli syndrome, *US* Ultrasound

### Diagnostic timelines

**Fig. 1:**
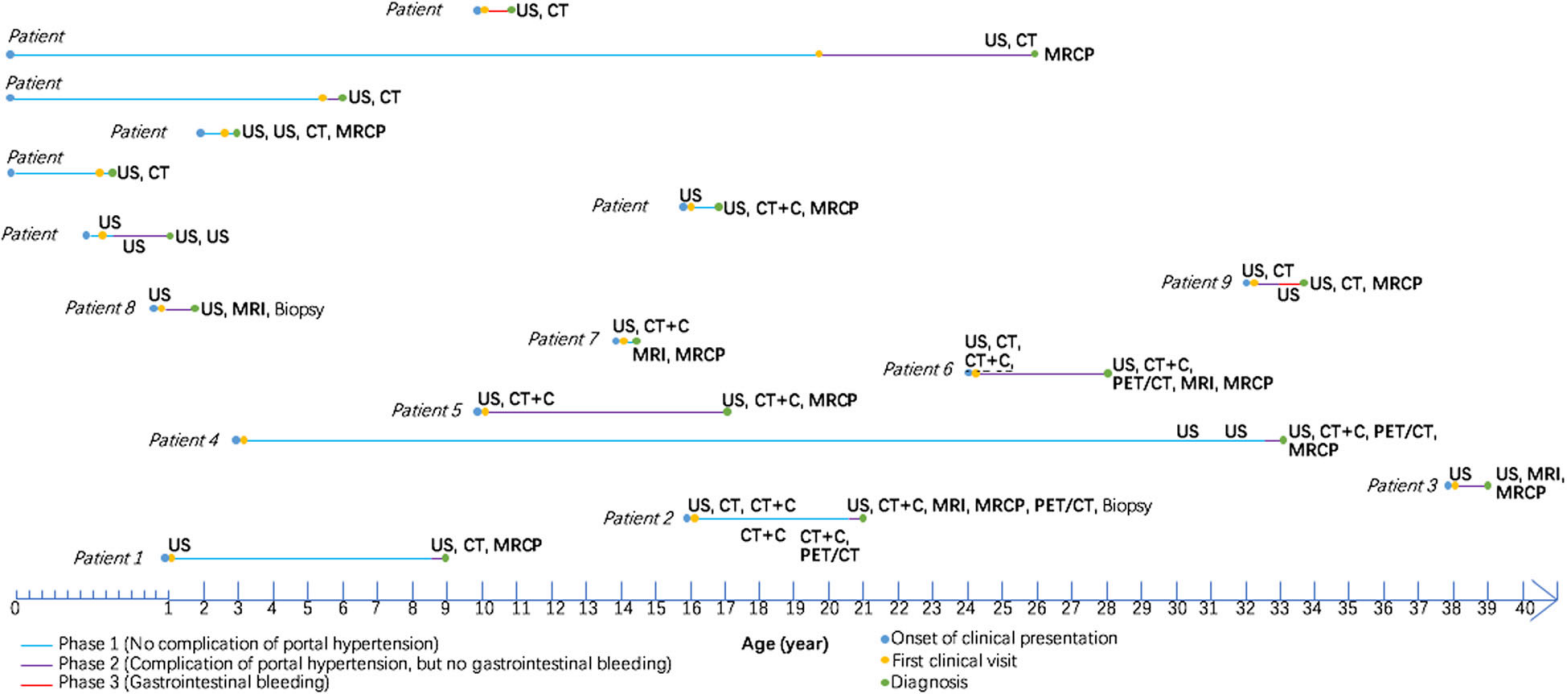
Diagnostic timelines of 16 CS patients. Important time points including onset of clinical presentation, first clinical visit, and diagnosis are depicted for each CS patient along the timeline. The course of the disease was divided into three phases: phase 1 (blue), defined as no proof of complication of portal hypertension (i.e., hypersplenism); phase 2 (purple), defined as discovered complication of portal hypertension without gastrointestinal variceal bleeding; and phase 3 (red), defined as at least one recorded variceal bleed. Imaging modalities and/or biopsy used before diagnosis are marked along the timelines. US: ultrasonography; CT: computed tomography; CT + C: computed tomography with contrast; MRI: magnetic resonance imaging; MRCP: magnetic resonance cholangiopancreatography; PET/CT: positron emission tomography/computed tomography

The diagnostic timelines of all 16 patients were drawn to visualize important diagnostic time points, disease phase at diagnosis, and imaging modalities used to make the diagnosis (Fig. 1). We divided the CS course into three phases: phase 1 (four patients, 25%), no proof of portal hypertension (i.e., hypersplenism); phase 2 (ten patients, 62.5%), discovered complications of portal hypertension without variceal bleeding; and phase 3 (2 patients, 12.5%), at least one recorded variceal bleed.

## Discussion

Caroli syndrome (CS) is a rare congenital disorder associated with ductal plate malformation and hepatic fibrosis [9]. CS is usually diagnosed during childhood or adolescence but may be diagnosed in adulthood [10, 11], consistent with our findings. Fifty percent of our CS patients developed symptoms before 6 years of age, and 18.75% of patients had symptom onset in adulthood. However, only 25% patients were correctly diagnosed before 6 years and 37.5% patients were not diagnosed until adulthood. The majority of patients already had complications of portal hypertension at the time of diagnosis and some even had severe gastrointestinal bleeding due to esophagogastric varices. There was also a wide range of diagnostic delay in our patients, from 1 month to as long as 29 years. However, to our best knowledge, there is no published evidence on the clinical factors influencing diagnostic delay in CS patients, indicating that diagnostic delay of CS patients might be a severe yet overlooked problem. We therefore attempted to analyze possible factors associated with diagnostic delay in CS patients to identify any clinical clues that could facilitate earlier diagnosis.

As previously reported, CS patients can present with fever and abdominal pain due to recurrent cholangitis, fatigue and ecchymosis due to hypersplenism, gastrointestinal bleeding due to varices, and non-specific digestive symptoms such as anorexia and abdominal distention [1, 4]. The most common initial symptoms in CS patients in our cohort were non-specific abdominal distention followed by fever, abdominal pain, variceal bleeding, and fatigue. Although three out of the eight patients initially presenting with abdominal distention did not seek immediate medical advice, which might have delayed the diagnosis of CS, there was no significant difference in diagnostic delay between patients with different initial symptoms.

Similarly, the laboratory findings in CS patients were not discriminative. Leukopenia, thrombopenia, and pancytopenia were associated with longer diagnostic delay. However, this was likely to be due to patients with longer diagnostic delay being more likely to have hypersplenism. Other laboratory abnormalities including increased ALT and bilirubin, decreased albumin, and prolonged PT were not associated with diagnostic delay. Interestingly, autoantibodies were positive in four (25%) of our CS patients, which has not previously been reported. Although the presence of these autoantibodies in AIH and PBC is well established, these autoantibodies are not reported to be associated with CS [12], which might therefore mislead the diagnosis. Indeed, these positive autoantibody results did lead to, or at least contribute to, the initial misdiagnosis of two CS patients. It is important to be aware of the co-existence of autoantibodies and CS, since immunosuppressive therapy might lead to clinical exacerbation of CS.

Imaging studies remain the primary diagnostic modality in patients with CS due to their non-invasiveness compared to liver biopsy, which when performed is usually to establish the degree of fibrosis [1, 4]. Although a range of *PKHD1* mutations are associated with CS, the diagnosis is usually made through the characteristic clinical picture, typical imaging features with or without histological confirmation, and exclusion of other hepatic diseases leading to hepatic fibrosis and bile duct dilatation. Most (87.5%) of our patients were diagnosed according to typical imaging features by US, CT, and MRI. The diagnosis of CS relies on demonstrating cystic dilatation of intrahepatic bile ducts in continuity with the biliary tree as well as signs of hepatic fibrosis. US features of the liver in CS include intrahepatic cystic anechoic areas in which fibrovascular bundles, stones, and linear bridging or septa may be present [1, 13]. Nevertheless, it is often difficult for radiologists to differentiate by US intrahepatic cysts caused by CS from cysts arising through other causes such as polycystic liver disease, and interobserver variability according to radiologist experience in making the diagnosis of CS has been reported [13]. In our study, the accuracy of diagnosing CS by US was only 25%, and some patients were not successfully diagnosed by US at first but were later diagnosed by another US performed in another hospital. Taking this interobserver variance into account, the actual accuracy of US for CS might be even lower.

**Fig. 2:**
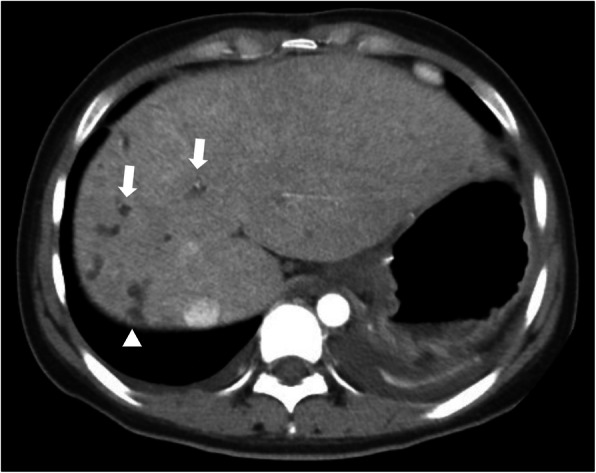
CT scan of a CS patient revealing multiple dilatations of intrahepatic bile ducts (arrow) and tiny dots of strong contrast within, which is the central dot sign (arrowhead)

**Fig. 3:**
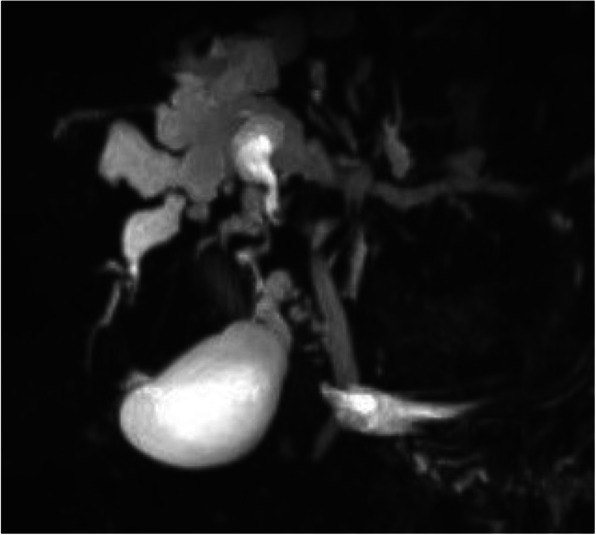
MRCP in a CS patient showing dilatation of intrahepatic bile ducts

In our study, CT scans were much more accurate than US (69.2%). The “central dot sign” on CT (Fig. 2), which refers to small foci of strong contrast enhancement within cystic lesions, is thought to correspond to portal radicles bridging dilatations and thus be pathognomonic of CS [14]. MRCP can establish the diagnosis of CS by revealing connections between bile duct ectasias and the normal biliary tract (Fig. 3) as well as ruling out other conditions like multiple liver abscesses and polycystic liver disease [13, 14]. The accuracy of MRCP was 83.3% in our study, similar to previously reported [5, 13]. In our study, the use of MRI/MRCP was limited, particularly in children. This was because: (i) MRI examination requires good patient cooperation, and it its often difficult for children to cooperate with the examination; (ii) patients are not usually sedated during MRI in China, even in tertiary hospitals, not least due to the limited resources of many patients; and (iii) CT scans are cheaper than MRI/MRCP, and CT scans are usually better covered by medical insurance than MRI/MRCP in most areas of China. Nevertheless, patients who had a CT scan performed due to physician or radiologist suspicion at the first hospital visited had a significantly shorter diagnostic delay. This was the only risk factor associated with diagnostic delay in our study, suggesting that a high index of suspicion for the disease might be the most important factor influencing diagnostic delay in CS, whose rarity often leads to poor awareness of the condition and thus misdiagnosis.

Due to the retrospective nature of the study, we were only able to locate the previous CT images in four patients (two CT and two contrast CT scans), with the longest interval of 4 years between previous and current CT scans. These scans reported non-specific findings such as “hepatic cyst”, “polycystic liver”, and “focal cystic mass of bile duct”, with some cases also reporting “dilatations of intrahepatic bile ducts”. These scans were compared by an experienced radiologist, and no significant differences were observed between the previous and current CT scans. This finding is consistent with our suggestion that the low index of suspicion for CS might be the most important factor leading to diagnostic delay. However, since these comparative imaging data are limited and the intervals between CT scans were relatively short, further studies are required to further investigate radiological misdiagnosis.

**Fig. 4:**
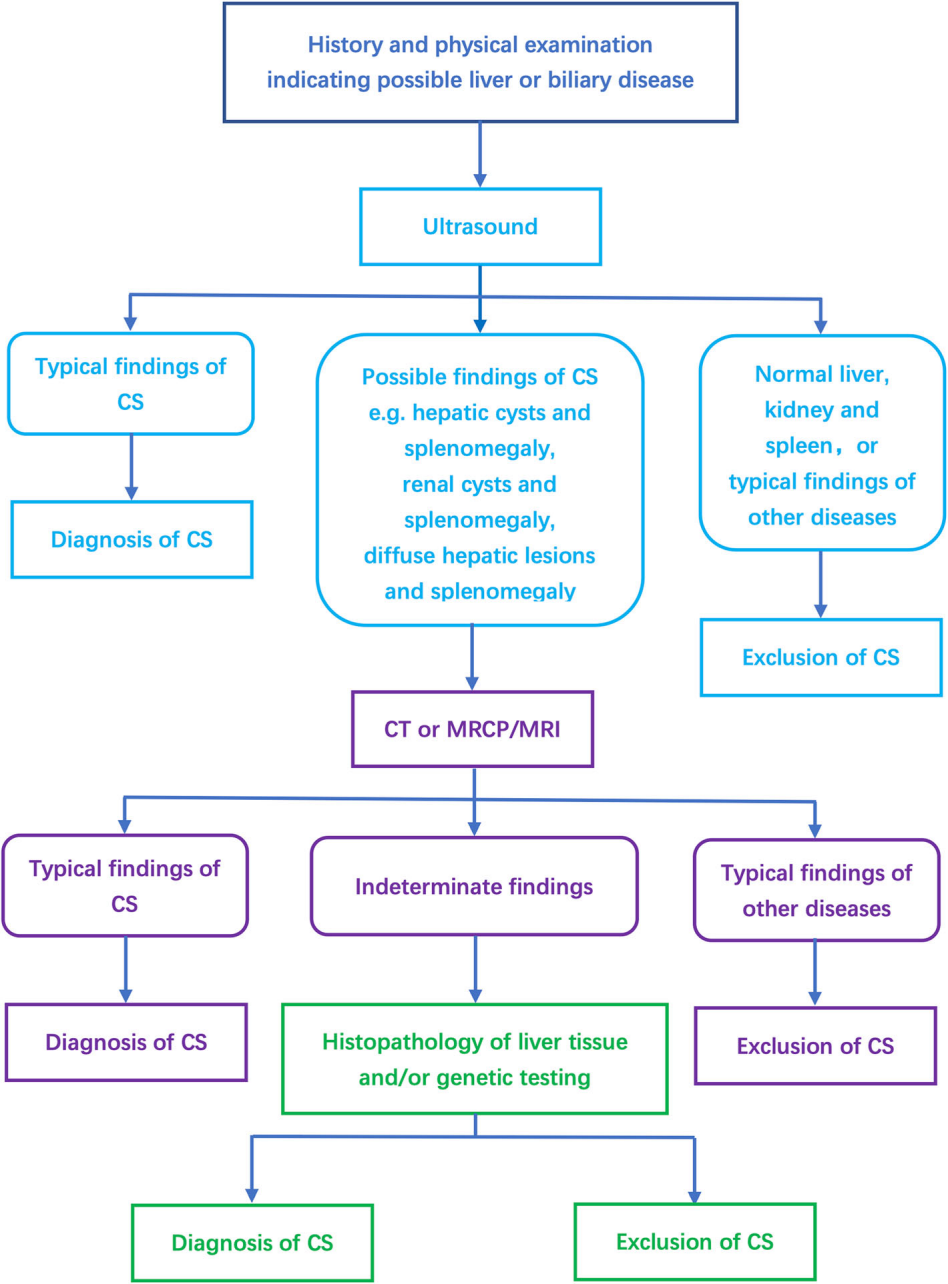
A proposed clinical practice workflow when there is a suspicion of CS

US was nearly always the first-line imaging modality due to its low cost and convenience, despite its suboptimal accuracy. Splenomegaly, diffusive hepatic lesions, and hepatic cysts were the most common US findings in CS. When combined, diffusive hepatic lesions plus splenomegaly and hepatic cysts plus splenomegaly were found in over half of our CS patients. However, diffusive hepatic lesions plus splenomegaly can also be found in a variety of other conditions including portal hypertension caused by thrombosis or dysplasia of the portal venous system, congenital disorders such as Niemann-Pick disease, congenital hepatic fibroses other than CS, and even malignancies like lymphoma [15]. Hepatic cysts with splenomegaly have a narrower differential diagnosis, mainly other hepatic cystic diseases including polycystic kidney disease [16], hepatic peribiliary cysts (which only rarely lead to splenomegaly) [17], and, even more rarely, malignant cystic diseases of the liver and spleen, which are easily differentiated [18]. Thus, hepatic cysts plus splenomegaly on US might provide a useful clue to physicians and thus might shorten the diagnostic delay of CS. Based on our results, Fig. 4 shows our proposed clinical practice workflow when CS is suspected.

This study has several limitations. First, the study was retrospective, and although we detected possible risk factors associated with diagnostic delay of CS patients, they require further verification. Second, since CS is rare the sample was relatively small, and the results require validation in larger samples. Third, we took the natural logarithm of diagnostic delay to make it normally distributed so that it could be compared using the *t*-test, which is more susceptible to outliers than the U test. Finally, we are a tertiary hospital and this is a single-center study, which could introduce bias in that patients sent to our hospital might be more complicated and difficult to diagnose.

## Conclusions

CS can be insidious and has no distinguishing symptoms or laboratory findings. The majority of patients were not diagnosed until complications of portal hypertension had already developed. Some patients might have positive autoantibodies, which could be deceptive and lead to misdiagnosis. US was not ideally accurate, but hepatic cysts with splenomegaly detected by US might raise the diagnostic index of suspicion for CS. Early suspicion of the disease might be the most important factor influencing diagnostic delay of CS.

## Data Availability

All data supporting our findings can be found within the manuscript.

## Abbreviations

CS: Caroli syndrome
US: Ultrasonography
CT: Computed tomography
MRI: Magnetic resonance imaging
SD: Standard deviation
IQR: Interquartile ranges
ALT: Alanine aminotransferase
PT: Prothrombin time
ANA: Antinuclear antibodies
AMA-M2: Anti-mitochondrial antibodies (M2 subtype)
SMA: Anti-smooth muscle antibodies
AST: Aspartate transaminase
IgG: Immunoglobulin G
AIH: Autoimmune hepatitis
GGT: Gamma-glutamyltransferase
ALP: Alkaline phosphatase
WBC: White blood cell
PLT: Platelet
MRPC: Magnetic resonance cholangiopancreatography
PBC: Primary biliary cholangitis
CT + C: Computed tomography with contrast
PET/CT: Positron emission tomography/computed tomography
Ln: Natural logarithm
DD: Diagnostic delay
HGB: Hemoglobin
